# Spinal cord‐predominant neuropathology in an adult‐onset case of *POLR3A* ‐related spastic ataxia

**DOI:** 10.1111/neup.12775

**Published:** 2021-11-09

**Authors:** Trevor M. Sytsma, Dong‐Hui Chen, Bradley Rolf, Michael Dorschner, Suman Jayadev, C. Dirk Keene, Jing Zhang, Thomas D. Bird, Caitlin S. Latimer

**Affiliations:** ^1^ Neuropathology Division, Department of Laboratory Medicine and Pathology University of Washington School of Medicine Seattle Washington USA; ^2^ Department of Neurology University of Washington School of Medicine Seattle Washington USA; ^3^ Division of Medical Genetics, Department of Medicine University of Washington School of Medicine Seattle Washington USA; ^4^ Geriatric Research Education and Clinical Center VA Puget Sound Health Care System Seattle Washington USA

**Keywords:** hereditary ataxia, Hereditary spastic paraplegia, POLR3A, spastic ataxia, spinal cord

## Abstract

Biallelic mutations in *POLR3A* have been associated with childhood‐onset hypomyelinating leukodystrophies and adolescent‐to‐adult‐onset spastic ataxia, the latter of which has been linked to the intronic variant c.1909 + 22G>A. We report a case of adult‐onset spastic ataxia in a 75‐year‐old man, being a compound heterozygous carrier of this variant, whose brain and spinal cord were for the first time investigated by neuropathological examination. We describe prominent degeneration of the posterior columns, spinocerebellar tracts, and anterior corticospinal tracts of the spinal cord in a pattern resembling Friedreich's ataxia, with a notable lack of significant white matter pathology throughout the brain, in marked contrast with childhood‐onset cases. Immunohistochemical examination for the POLR3A protein demonstrated no apparent differences in localization or staining intensity between the proband and an age‐matched control subject. We demonstrate the clinicopathologic description of *POLR3A*‐related neurodegenerative disease and also mention the differential diagnosis of the childhood‐onset hypomyelinating leukodystrophy and late‐onset spastic ataxia phenotypes.

## INTRODUCTION

POLR3‐related leukodystrophies are a rare class of autosomal recessive neurogenetic disorders clinically characterized by various combinations of neurological motor, extrapyramidal, pyramidal, and cognitive dysfunctions, abnormal dentition, endocrine abnormalities (e.g. short stature and hypogonadotropic hypogonadism), and myopia.^1^ These leukodystrophies result from a number of biallelic pathogenic variants in *POLR3A*, *POLR3B*, *POLR1C*, and *POLR3K*, which encode individual subunits of the RNA polymerase III (Pol III) complex. This complex is responsible for synthesizing several noncoding RNAs, including tRNAs, 5S RNA, 7SK RNA, U6 RNA, and mitochondrial RNA‐processing RNA, that regulate critical cellular processes^2^ and functions as a cytosolic DNA sensor involved in innate immune responses.^3^

The clinical, radiographic, and molecular characteristics of *POLR3*‐related disorders have been well‐described.^4^, ^5^, ^6^, ^7^, ^8^, ^9^ While the most recognized phenotypes are characterized by a hypomyelinating leukodystrophy with early‐childhood onset, recent reports suggest that a less severe spastic ataxia phenotype with adolescent‐to‐adult onset, without hypomyelination, may be associated with an intronic mutation (c.1909 + 22G>A) in *POLR3A*.^10^, ^11^, ^12^, ^13^, ^14^, ^15^, ^16^ There is still debate regarding the mutation's specificity for this clinical phenotype,^16^, ^17^, ^18^, ^19^ and there are no precedent neuropathological reports of the adolescent‐to‐adult‐onset form of this disease. Here, we report the clinical course and neuropathologic features of the brain and spinal cord from a 75‐year‐old man with adult‐onset spastic ataxia, being a compound heterozygous *POLR3A* carrier.

## CLINICAL SUMMARY

The patient was the third of four sons born to non‐consanguineous parents. His father, who was reported to walk with a cane for unknown reasons, died in an automobile accident at the age of 35 years. His mother had no history of neurological impairment. The patient's childhood and early adolescence were unremarkable, and he met normal developmental milestones. While in the military at age 19, he was first diagnosed as having an unknown type of neurological disability, primarily characterized by an abnormal, unsteady gait. Despite increasing motor impairment, he retained the ability to perform delicate tasks with his hands while working in an electronics assembly plant up to his retirement around age 50. However, due to slowly progressive ataxia and incoordination, he was eventually seen by neurologists at the VA Puget Sound Medical Center in Seattle at age 52, where he was noted to be walking with arm crutches. Physical examination revealed pyramidal signs (i.e. spasticity of lower extremity muscles, increased deep tendon reflexes, ankle clonus, and bilateral Babinksi responses), cerebellar signs (i.e. slight horizontal nystagmus on lateral gaze, dysarthria, dysmetria, and mild head titubation), and symmetric peripheral neuropathy (i.e. distal sensory loss in both feet of pain and vibration), leading to a diagnosis of spinocerebellar degeneration. A follow‐up examination four years later revealed bilateral delay in median nerve somatosensory‐evoked potentials (SEP). His condition continued to progress, and he became dependent on a wheelchair but otherwise continued to manage all activities of daily living while living alone.

Magnetic resonance imaging (MRI) of the brain performed at age 67 revealed neither white matter abnormality nor cerebellar atrophy. At about the same age, neurological examination redomonstrated previous findings and identified decreased of deep tendon reflexes with no evidence of nystagmus. Electrophysiologic studies revealed absent sensory responses of the sural nerve and decreased nerve amplitudes evoked by median and ulnar sensory stimulation and by peroneal motor stimulation. Concentric needle electromyography revealed fibrillations and positive sharp waves in the anterior tibialis. Large amplitude motor units with decreased recruitment pattern were seen in the anterior tibialis and vastus medialis. Cumulatively, these results provided electrodiagnostic evidence of a mild, axonal sensorimotor peripheral polyneuropathy.

Throughout life, his cognitive abilities remained intact, and gonadal dysfunction was never identified. He was prescribed baclofen to help manage his symptoms. He died at age 75 of post‐obstructive pneumonia due to an enlarging non‐small cell carcinoma of the lung. He had two sons who lived into adulthood without neurological problems.

The patient's younger brother presented with a progressive but less severe spastic ataxia at age 15, developing symptoms, including pyramidal and cerebellar signs and peripheral neuropathy, similar to those of the proband. He died by suicide at age 63. He had one now‐deceased child with a diagnosis of pseudotumor cerebri.

## ETHICS PROCEDURE FOR POSTMORTEM EXAMINATION

Informed consent for research brain donation was obtained from the legal next of kin according to protocols approved by the University of Washington Institutional Review Board that conform to the provisions of the Declaration of Helsinki and preserve patient anonymity.

## PATHOLOGICAL FINDINGS

### Macroscopic features

The brain and spinal cord were removed 24 h after death. The fresh whole brain weighed 1310 g, displayed a normal mature gyral pattern but no significant cortical atrophy. of the cerebral cortex, and was processed with immersion fixation in 10% neutral‐buffered formalin for at least two weeks. The fixed whole brain weighed 1410 g and was observed externally and photographed. Then, the cerebellum and brainstem were separated from the cerebrum by axial transection at the level of the oculomotor nerves of the midbrain. The brainstem and cerebellum weighed 140 g. The cerebrum was sliced coronally at a thickness of 0.5–1‐cm. At a thickness of approximately 5 mm, the brainstem and spinal cord were individually sliced axially, and the cerebellum was sliced along the parasagittal plane. Gross examination was performed by a board‐certified neuropathologist (J.Z.) for developmental anomalies, focal lesions, and other abnormalities. Examination of the whole brain and brain slabs revealed none of the focal cavitary, massive, or hemorrhagic lesions or no significant evidence of brain herniation, hippocampal atrophy, or discoloration of the basal ganglia. Cerebrovascular disease changes were as expected for age, and the subcortical white matter volume was not obviously reduced or without otherwise abnormal alteration. The brainstem, cerebellum, and spinal cord were grossly unremarkable, although the transverse diameter of the cervical spinal cord was slightly reduced at 9.6 mm. Tissue specimens of the relevant regions from the brain and spinal cord were processed on an ASP6025 automated tissue processor (Leica Biosystems, Buffalo Grove, IL) according to standard protocols and then embedded in paraffin for microscopic examination.

### Microscopic features

**Fig. 1:**
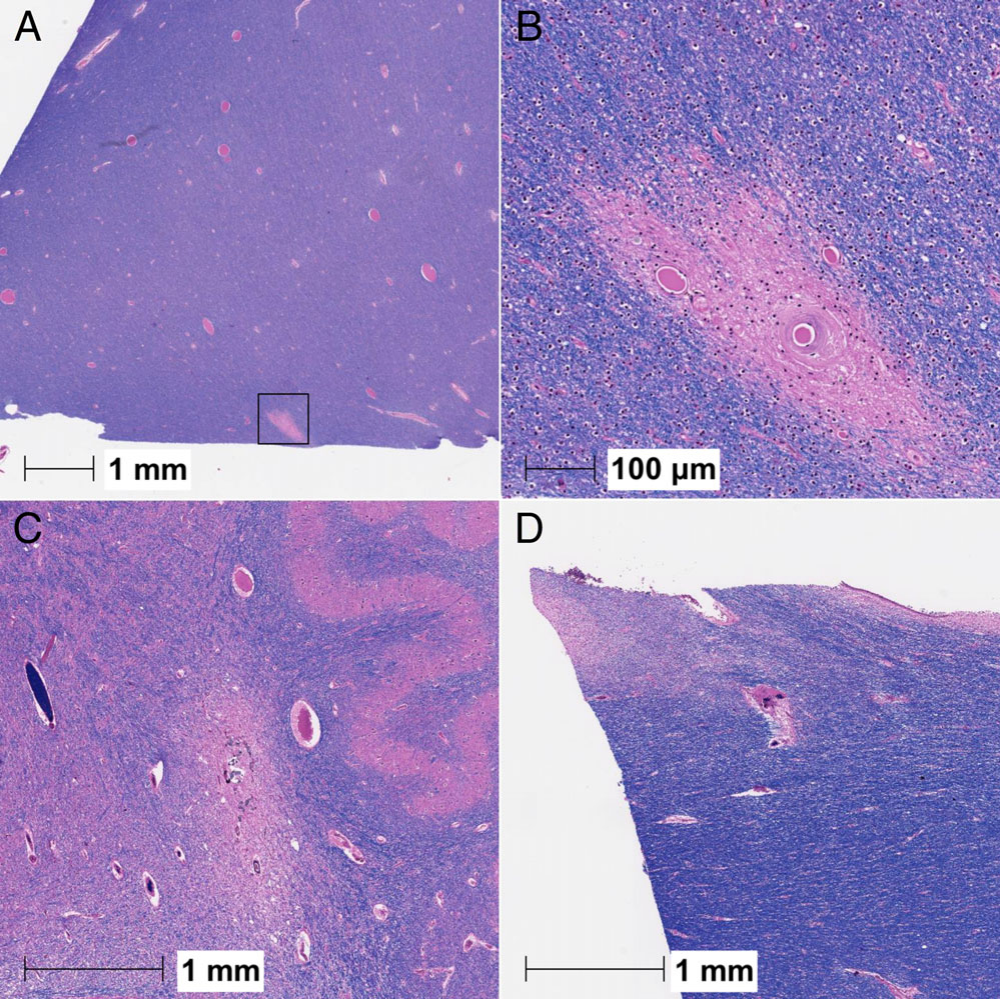
Histological features of the brain white matter. (A–D) Perivascular white matter lesions and patchy areas of myelin loss are observed in some regions but are not widespread throughout the brain. Myelin is well‐preserved throughout the subcortical white matter of the cerebrum (A). Panel (B) displays a higher magnification view of a square area of panel (A). White matter lesions, although sparse, are frequently observed in close proximity to small blood vessels with age‐related arteriolosclerosis (B). Perivascular white matter lesions are observed near the inferior olivary nucleus of the medulla oblongata (C). Focal myelin pallor is found in a superior and medial portion of the corpus callosum at the level of the anterior commissure (D) HE/LFB staining (A–D).

Multiple 5‐μm‐thick sections of formalin‐fixed, paraffin‐embedded (FFPE) tissue blocks were stained with hematoxylin and eosin/Luxol fast blue HE/LFB. Immunohistochemistry was performed by previously optimized protocols using a Leica BOND‐MAX Fully Automated IHC and ISH staining system (Leica Biosystems, Buffalo Grove, IL, USA). HE/LFB‐stained sections showed that sampled subcortical white matter regions including tracts were generally well‐preserved throughout the brain (Fig. 1A). Rare foci of age‐related perivascular alteration were present in the frontal lobe (Fig. 1B) and near the inferior olivary nucleus of the medulla oblongata (Fig. 1C). A small focus of myelin loss was noted in the midline of the corpus callosum at the level of the anterior commissure (Fig. 1D). The white matter was otherwise unremarkable throughout the cerebrum and brainstem.

The brainstem was evaluated at several levels and overall was well‐formed, without evidence of atrophy. A number of brainstem nuclei were examined and appeared unremarkable, with no significant neuronal loss. These nuclei included the substantia nigra in the midbrain, the locus ceruleus and pontine nuclei in the pons, and the inferior olivary nucleus, the hypoglossal nucleus, the vestibular and cochlear nuclei, the dorsal nucleus of the vagus nerve, and the arcuate nucleus in the medulla oblongata. The white matter tracts traversing the brainstem also appeared normal and exhibited no significant myelin pallor or atrophy. These included the pyramidal tracts at all levels, the crossing pontocerebellar fibers in the pons, and a portion of the superior cerebellar peduncles, observed in the dorsolateral pons.

**Fig. 2:**
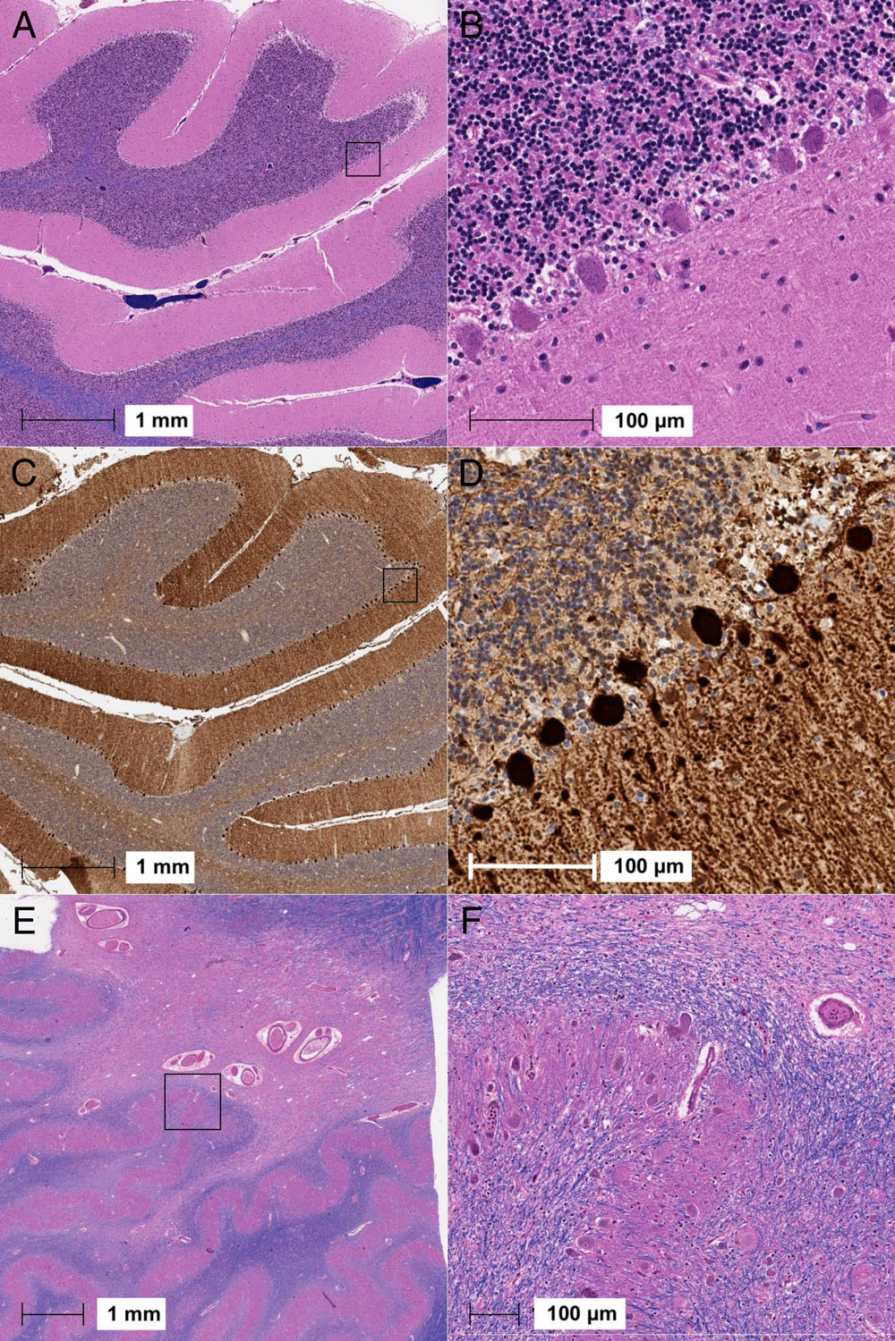
Microscopic features of the cerebellum. Only focal myelin pallor is observed. (A) The cerebellar cortex shows no significant alteration. (B) Purkinje cells are present in an expected density and show no significant gliosis, atrophy of the molecular layer, or Purkinje cell dropout. (C, D) Calbindin D‐28k immunoreactivity is preserved in the neuropil and Purkinje cells of cerebellar cortex. (E) Myelin pallor is seen in the deep cerebellar white matter with no significant alteration of adjacent dentate nucleus, characterized by the lack of neuronal loss, gliosis, and neuropil rarefaction. Panel (F) indicates a higher magnification view of panel (E). HE/LFB staining (A, B, E, F), immunohistochemical staining (C, D).

The cerebellar cortex was normal, with well‐formed folia with no significant atrophy or gliosis. There was no significant evidence of Purkinje cell loss or atrophy, confirmed on HE/LFB staining and immunohistochemistry with a mouse monoclonal anti‐calbindin D‐28k antibody (clone CB‐955; Sigma‐Aldrich, St. Louis, MO, USA; 1:1000) (Fig. 2A–D). No so‐called “empty baskets” were observed on Bielschowsky silver impregnation (data not shown). The dentate nucleus showed no significant diagnostic change, evidenced by no notable neuronal loss, gliosis, or rarefaction of the neuropil. However, the hilar outflow in the surrounding deep cerebellar white matter showed significant myelin pallor. (Fig. 2E, F).

**Fig. 3:**
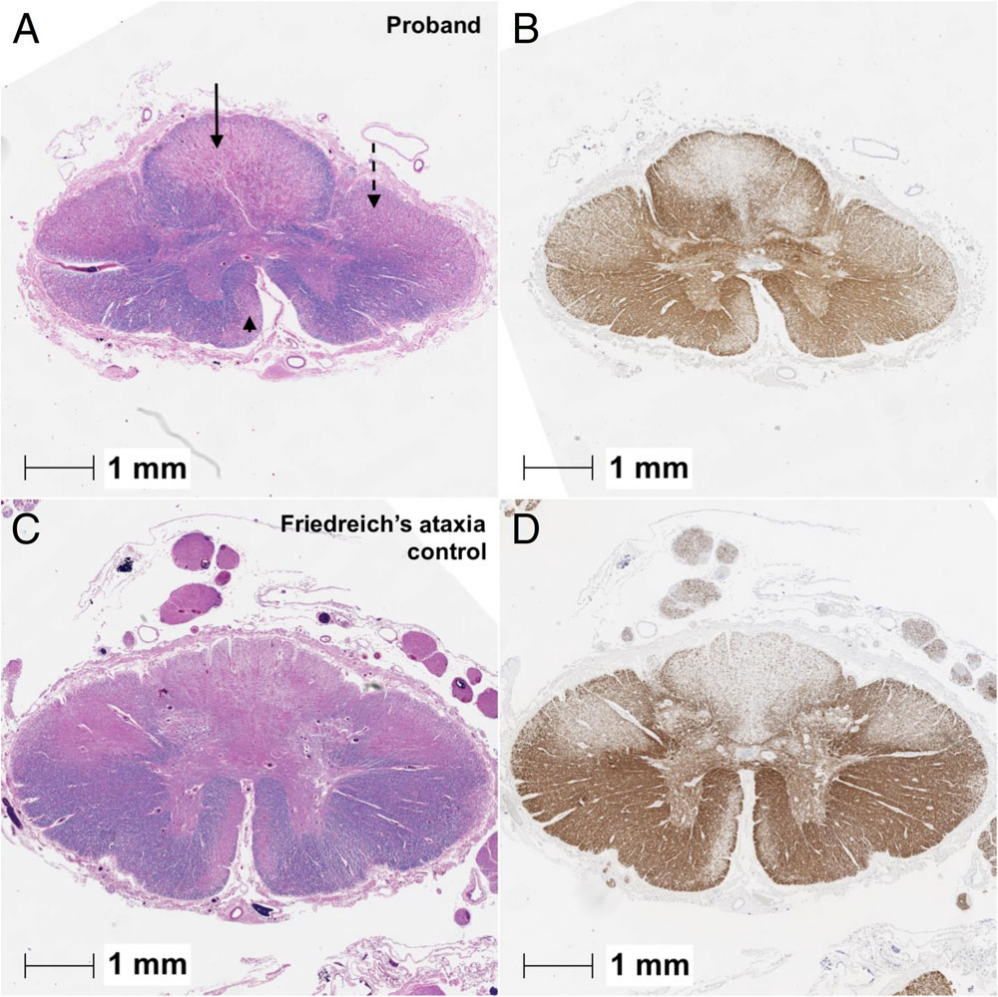
Semimacroscopic features of the spinal cord. (A, B) In consecutive sections of a lower thoracic segment from the proband, morphological changes in the dorsal columns (arrow), spinocerebellar tracts (dotted arrow), and ventral corticospinal tracts (arrowhead) resemble degeneration seen in Friedreich's ataxia, demonstrated on HE/LFB staining (A) and MBP immunohistochemistry (B). (C, D) Consecutive sections of a lower thoracic segment from an age‐, sex‐, and postmortem interval‐matched patient with Friedreich's ataxia, demonstrated on HE/LFB staining (C) and MBP immunohistochemistry (D).

In the spinal cord, the neurons in the anterior horns and Clarke's columns were intact; nuclear or cytoplasmic inclusions immunoreactive with a mouse monoclonal anti‐ubiquitin antibody (clone Ubi‐1; Millipore, Burlington, MA, USA; 1:500) were undetectable in the ventral nerve roots of the lumbar segments. However, there was evidence of a severe reduction in tissue volume and immunoreactivity with a mouse monoclonal anti‐myelin basic protein (MBP) antibody (clone MBP101; ab62631, Abcam, Cambridge, UK; 1:1000) in multiple ascending and descending pathways in the cervical, thoracic, and lumbar segments, including the spinocerebellar tracts and dorsal columns as well as the anterior and lateral corticospinal tracts, in a pattern very similar to those in Friedreich's ataxia (Fig. 3A, B).^20^ On HE/LFB staining, the spinal cord showed no significant perivascular lymphocytic or macrophage infiltration or demyelination. Only very limited portions of peripheral nerves were present in sections of the spinal cord, and, although rare, some foci of myelin pallor were observed; the limited sampling precludes a thorough evaluation and definitive assessment of the peripheral nerves. Histological and immunohistochemical examination performed in a 66‐year‐old and postmortem interval‐matched subject with Friedreich's ataxia revealed nearly identical white matter myelin pallor patterns (Fig. 3C, D). As in Friedreich's ataxia, we interpret these findings to represent selective loss of axons in certain spinal motor and sensory pathways.

**Fig. 4:**
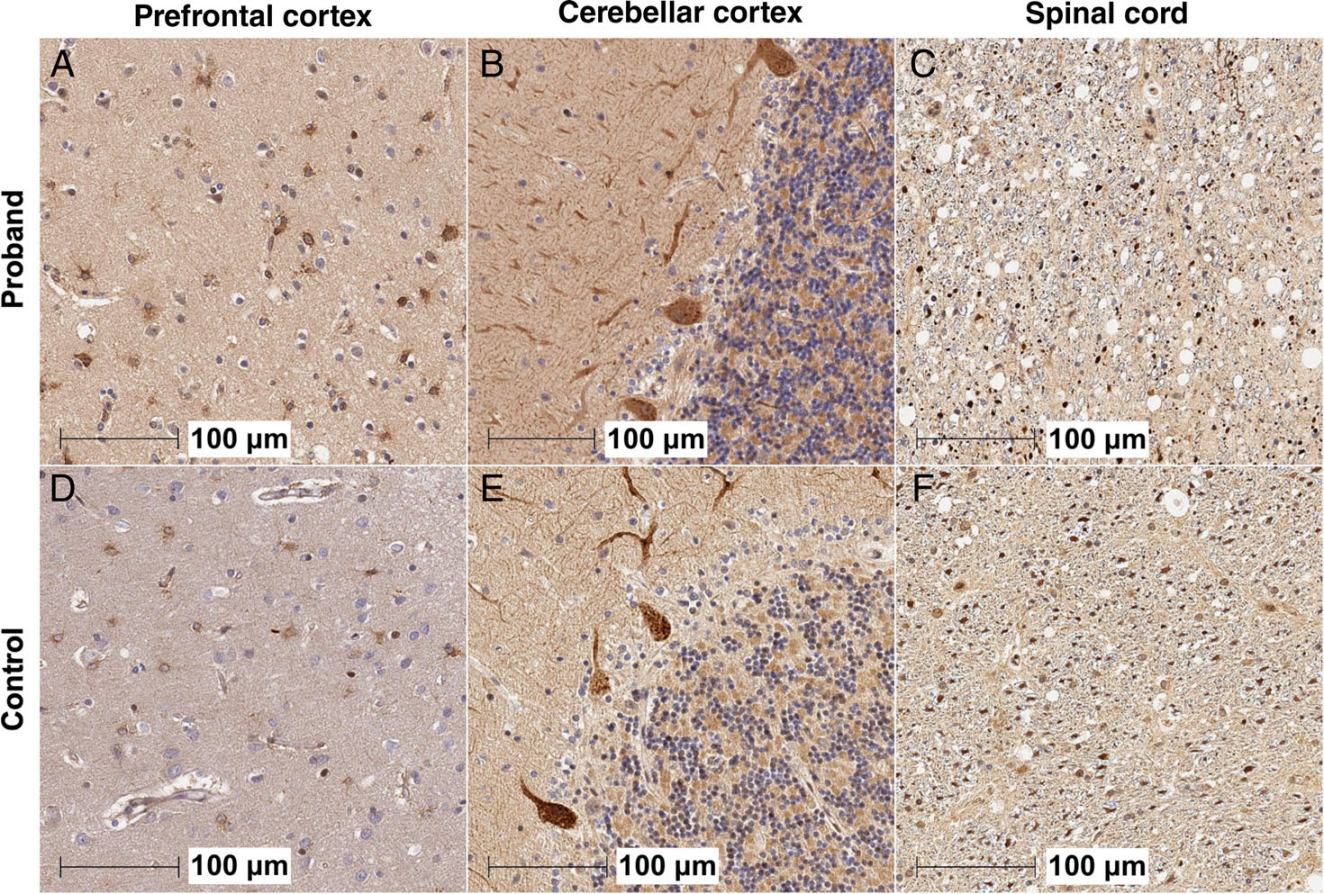
Immunohistochemical features for POLR3A in the brain. There is no apparent difference in POLR3A staining patterns between the proband and an age‐, sex‐, and postmortem interval‐matched control subject. (A, D) POLR3A immunoreactivity is ubiquitously distributed and shows variable intensity in cell nuclei of neurons and glia in the prefrontal cortex of both the proband and the control. (B, E) POLR3A immunoreactivity highlights somata and dendrites of Purkinje cells in the cerebellar cortex of both the proband and the control. (C, F) Oligodendrocytes exhibit nuclear staining for POLR3A in the ventral corticospinal tracts of the spinal cord of the proband and the control.

After uncovering variants in the proband's *POLR3A* genotype, additional immunohistochemical protocols were developed for this study to assess expression and localization of POLR3A in tissue sections from the proband and a sex‐, age‐, and postmortem interval‐matched control procured within the same 18‐month period using a mouse monoclonal anti‐POLR3A antibody (clone PA5‐58170; Thermo Fisher Scientific, Waltham, MA, USA; 1:200). POLR3A immunohistochemistry revealed both nuclear and cytoplasmic staining in the proband and control, indicative of expression of POLR3A as a cytosolic DNA sensor (Fig. 4A–F). Nuclear staining of variable intensity was observed in both neurons and glia in the frontal cortex. POLR3A immunoreactivity also highlighted somata and dendrites of Purkinje cells. Notably, the immunoreactivity throughout multiple areas of the proband's spinal cord was indistinguishable from that observed in the control.

## GENETIC FINDINGS

### Antemortem genetic workup

During his life, the patient underwent extensive targeted testing for known genes associated with ataxia and spasticity (spastic paraplegias 3A, 4, and 7; spinocerebellar ataxias 1, 2, 3, 6, 7, and 14; *FXN‐*, *APTX‐*, *SETX‐*, *PLP1‐*, and *LCFA*‐related disorders). His serum levels of homocysteine, vitamins B_12_ and E were also measured. All test results were normal.

### Postmortem exome sequencing

The Northwest Clinical Genomics Laboratory (NCGL), a CLIA‐certified laboratory at the University of Washington, conducted exome sequencing for the proband and his similarly affected brother. Gene libraries were assembled according to an in‐house protocol developed by the NCGL. The NCGL used a KAPA Hyper Prep DNA library kit (KAPA Biosystems, Wilmington, MA, USA) to prepare the libraries, which were subsequently enriched using an in‐house optimized xGen Exome Research Panel v1.0 (Integrated DNA Technologies, Coralville, IA, USA). The mean depth of coverage was 152.21 reads, and an average of 99.55% of the captured target sequences was covered at a depth of at 20‐fold or greater. Variants were identified using the Genome Analysis Toolkit (GATK)^21^, ^22^ and annotated using SnpEff tools^23^ in combination with various population databases and variant impact scoring tools.

Postmortem exome screening ultimately uncovered two known pathogenic variants in *POLR3A*: c.1051C>T and c.1909 + 22G>A. The former coding region variant forms a premature stop codon (p.Arg351*),^7^ whereas the latter intronic variant activates a leaky splice site that causes inclusion of 19 nucleotides of intron 14, leading to a shift in the reading frame and a premature stop codon following 13 extraneous amino acids (p.Y637Cfs^*^14).^10^ Identical variants were identified in a banked DNA sample from his similarly affected, deceased younger brother.

### Postmortem evaluation of *POLR3* transcript expression

In order to compare expression levels of *POLR3* transcripts, we performed reverse transcription‐polymerase chain reaction (RT‐PCR) analysis using total RNA extracted from FFPE inferior parietal lobe sections from the proband and a normal control used for POLR3 immunohistochemistry comparison. Total RNA was extracted from 10‐μm‐thick FFPE sections using a PureLink FFPE RNA Isolation Kit (Thermo Fisher Scientific) according to the manufacturer's protocol. To obtain RNA of satisfactory quality, several modifications,, such as varying the amount of starting material and increasing the heating time and temperature for proteinase K digestion, were attempted. RNA extraction was also attempted using a High Pure FFPE RNA Micro Kit (Roche Life Science, Penzberg, Germany). RNA abundance and quality were analyzed using a Qubit RNA HS Assay Kit (Thermo Fisher Scientific). An aliquot of 400 ng of RNA was reverse transcribed to cDNA using a SensiFast cDNA Synthesis Kit (Bioline, London, UK) with *POLR3A*‐specific primers in addition to a random primer. Amplification of an exon 14–15 *POLR3A* fragment encompassing the variant c.1909 + 22G>A, an exon 7–9 fragment encompassing the variant c.1051C>T, and an exon 2–3 fragment upstream of these variants was carried out on the cDNA, derived from 200 ng of RNA, using HotStarTaq (Qiagen, Hilden, Germany). In an effort to enhance the sensitivity and specificity of amplification, nested PCR was performed using two successful available primer sets. We also tried one‐step RT‐PCR to specifically synthesize and amplify *POLR3A* cDNA fragments directly from RNA. In all PCR amplifications, RNA extracted from lymphoblastoid cell line (LCL) cells of a normal control was included as a control. The house‐keeping glyceroaldehyde 3‐phosphate dehydrogenase (GAPDH) gene *GAPDH* was amplified as an internal control of RNA/cDNA quality. We were able to amplify short *GAPDH* fragments in both subjects; however, amplification of *POLR3A* fragments was unsuccessful.

## DISCUSSION

*POLR3A*‐related disorders are characterized by a broad range of onset ages, clinical symptoms, and phenotype–genotype associations, suggesting that certain variants are related to different forms of the disease. Adolescent‐to‐adult‐onset spastic ataxia linked to compound heterozygosity for an intronic mutation (c.1909 + 22G>A) in *POLR3A* has now been reported in several individuals and families.^10^, ^11^, ^12^, ^13^, ^14^, ^15^, ^16^ This phenotype does not recapitulate the diffuse hypomyelinating leukodystrophy observed in childhood‐onset cases; however, MRI often reveals hyperintensity of the superior cerebellar peduncles and variable atrophy of the spinal cord, cerebellum and corpus callosum. In this report, a man with adult‐onset spastic ataxia was found to have two pathogenic variants in *POLR3A*: c.1051C>T (p.Arg351*) and c.1909 + 22G>A (p.Y637Cfs^*^14). We provide the first neuropathological evidence to support the role of these variants in the pathogenesis of spastic ataxia and expand the phenotypic description of *POLR3A*‐related disorders.

The proband exhibited many of the clinical features of adolescent‐to‐adult‐onset spastic ataxia, including sensory loss, abnormal SEP, lower limb spasticity, muscle atrophy, dysarthria, and polyneuropathy, associated with the presence of the c.1909 + 22G>A allele.^13^ Notably, he showed the lack of signs of non‐neurological features common in *POLR3*‐related disorders, such as abnormal dentition, endocrine abnormalities, and myopia, and, the age of 75 years at death may be the longest‐living individual.

The absence of diffuse hypomyelination is a key feature distinguishing adult‐onset form from childhood‐onset form of *POLR3A*‐related disorders. Indeed, neuropathological examination of this case revealed generally normal myelination throughout the cerebrum and brainstem, with the exception of a few foci of perivascular white matter rarefaction. The characteristic MRI feature described in adult‐onset cases, particularly those carrying the c.1909 + 22G>A allele, is hyperintensity of the superior cerebellar peduncles extending from the dentate nucleus up to the midbrain.^11^, ^15^ Although only a small portion of the patient's superior cerebellar peduncles was available for examination due to the fact that the brain and spinal cord were initially sampled, processed, and reviewed without knowledge of his *POLR3A* carrier status, we did note a focus of severe myelin pallor of the hilar outflow tract of the deep cerebellar white matter. Given the patient's age and the presence of vascular pathology, we cannot exclude the possibility that this white matter lesion was based on age‐related vascular disease; however, the involvement of fiber tracts that compose the superior cerebellar peduncle in the context of a c.1909 + 22G>A allele highly suggests that this is, indeed, a component of the *POLR3A*‐related pathology. Furthermore, despite the limited pathology observed, the location of this lesion suggests that it is related to some of the symptoms, including dysarthria and nystagmus. These symptoms may also be due to lesions in other parts of the cerebellum or brainstem. Therefore, we cannot exclude pathological changes in unsampled regions.

Spinal cord neuropathology has been reported in one other patient with a form of *POLR3A*‐related disorder to date; patchy areas with myelin loss in a patient with pediatric‐onset leukodystrophy were described.^7^ Spinal cord atrophy, detected by MRI, is characteristic of nearly all reported adolescent‐to‐adult‐onset cases carrying c.1909 + 22G>A allele. In the current case, we describe severe degeneration of multiple white matter tracts extending from cervical through lumbar segments of the spinal cord, including posterior columns and long motor tracts. Both loss of myelin and axons was observed, and it was not clear from the histology alone whether this represented a primary axonopathy or a primary myelinopathy. The pathology closely resembles that of Friedreich's ataxia and is consistent with the many neurological signs overlapping these conditions, including gait ataxia, dysarthria, sensory loss, atrophy and weakness of distal extremity muscles, polyneuropathy, lower limb spasticity, and head titubation.^20^ There is increasing speculation that clinical overlap along the ataxia‐spasticity disease spectrum is driven by mechanistic overlap and shared susceptibility to disturbances in common molecular pathways. Indeed, in protein–protein interaction networks proposed by Synofzik and Schüle, the proteins encoded by *POLR3A* and *FXN*, as the causative gene of Friedreich's ataxia, were shown to cluster in the same highly connected “protein community.”^24^ It is possible, for example, that the proteins and metabolic pathways affected by these two diseases encompass a common element involved in mitochondrial function. While the present report demonstrates some shared clinical and neuropathological features of late‐onset, *POLR3A*‐related spastic ataxia and Friedreich's ataxia, there are also some notable differences. For example, although the superior cerebellar peduncle shows the damage in both the diseases likely due to degeneration of neurons within the dentate nucleus, a finding not observed in *POLR3A‐*related spasticity. Larger studies are needed to validate and better characterize these findings in additional patients as well as assess the mechanistic overlap.

Based on the truncating *POLR3A* variants carried by the proband and our selection of an antibody specific to POLR3A amino acid residues 607–698, we hypothesized that immunohistochemistry would reveal reduced POLR3A staining. However, instead we identified similar staining patterns and intensity in the proband compared to a non‐affected control. In an effort to understand results of POLR3A immunohistochemistry and expression levels of *POLR3A* transcript in the brain, we attempted to extract RNA from FFPE inferior parietal lobe sections (frozen tissue was unavailable) in the proband and the normal control and quantify wild‐type and variant *POLR3A* transcripts. While we were able to amplify short *GAPDH* fragments in both subjects, amplification of *POLR3A* fragments was unsuccessful despite modifications in RNA isolation and cDNA synthesis methodology and application of nested PCR with various primer sets. We attribute these difficulties to RNA degradation/inaccessibility associated with prolonged fixation, tissue processing, and time.

The transcript resulting from the c.1051C>T variant is also predicted to undergo nonsense‐mediated mRNA decay (NMD), although this awaits experimental confirmation. In addition, previous RNA expression studies using whole blood by Minnerop *et al*. showed that the aberrant c.1909 + 22G>A *POLR3A* transcript is of low abundance relative to wild‐type transcript due to both NMD and incomplete activation of the cryptic splicing site. Indeed, in silico scoring tools predict that wild‐type splicing is favored over splicing at the cryptic site (0.91 *vs* 0.74 by the Berkeley Drosophila Genome Project; 0.93 *vs* 0.82 by NetGene2), producing both wild‐type and truncated proteins.^25^, ^26^ Minnerop *et al*. investigated the effect of c.1909 + 22G>A and other truncating variants on POLR3A levels and found that leukocytes from patients who were compound heterozygous for c.1909 + 22G>A displayed only 60–80% of the total protein levels of those detected in wild‐type patients by Western blot analysis. We suspect that the retained immunohistochemical positivity and similar staining intensity observed in the proband and control tissues indicate this preserved wild‐type protein and the insufficient immunohistochemical sensitivity to resolve reductions in protein levels of this moderate magnitude. We also hypothesize that the remaining levels of wild‐type protein preserved by incomplete inactivation of the normal splicing site by c.1909 + 22G>A might underly the shift from the hypomyelinating leukodystrophy phenotype towards the spastic ataxia phenotype observed in the proband and other patients who are compound heterozygous carriers of this variant. Further studies of RNA transcript and protein expression in central nervous system tissue from similarly affected individuals are needed to provide more direct and accurate information about the effect of the c.1051C>T and c.1909 + 22G>A variants on disease pathogenesis.

In summary, we describe the clinical course of a 75‐year‐old man with compound heterozygosity in *POLR3A* for the c.1909 + 22G>A allele, which has been associated with an adolescent‐to‐adult‐onset spastic ataxia. We report novel neuropathological features of the phenotype, including spinal cord degeneration, POLR3A immunohistochemistry, and absence of diffuse white matter disease. These findings further characterize the spectrum and demonstrate the need for additional neuropathological evaluation of *POLR3A*‐related disorders.

## DISCLOSURE

The authors declare no conflict of interests for this article.
